# OPPORTUNITIES AND BARRIERS IN INTEGRATING PALLIATIVE CARE SERVICES INTO PERMANENT SUPPORTIVE HOUSING

**DOI:** 10.1093/geroni/igad104.1193

**Published:** 2023-12-21

**Authors:** Ian Johnson, Anthony Traver, Michael Light

**Affiliations:** University of Texas- San Antonio, San Antonio, Texas, United States; The Ohio State University, Columbus, Ohio, United States; University of Washington Palliative Care Training Center, Seattle, Washington, United States

## Abstract

Despite well-known inequities in rates of serious illness and premature mortality among people experiencing homelessness, as well as the unique biopsychosocial and spiritual care needs of people with homelessness histories, there is a lack of translational research focused on palliative care service delivery within permanent supportive housing. This presentation will present data from the Research, Action, & Supportive Care at Later-life for Unhoused People (RASCAL-UP study) to describe the current practices, perceived barriers, and recommendations in providing support to older permanent supportive housing residents during serious illness and end-of-life. Through a thematic analysis of interviews with direct care providers (n=30) across multiple care settings, themes were identified: 1) the importance of on-site relationships; 2) cross-sector collaboration barriers; 3) interdisciplinary communication barriers within permanent supportive housing teams; 4) environmental and spatial considerations; and 5) missing levels of care between supportive housing and higher levels of medical care. From these results, we put forth recommendations for translational research, supportive housing policy, and capacity-building efforts for interdisciplinary and cross-sector healthcare and housing teams.

